# Comparison of Blood Loss Between Volatile and Total Intravenous Anesthesia in Cesarean Delivery: A Single-Center Retrospective Observational Study

**DOI:** 10.7759/cureus.98750

**Published:** 2025-12-08

**Authors:** Kensuke Shimada, Mana Obata-Yasuoka, Masao Iwagami, Keitaro Kume, Takahiro Yano, Ryota Inokuchi, Kazushi Maruo, Hiroshi Ueda, Hiromi Hamada, Toyomi Satoh, Masaru Sanuki, Nanako Tamiya

**Affiliations:** 1 Department of Clinical Research and Regional Innovation, Institute of Medicine, University of Tsukuba, Tsukuba, JPN; 2 Translational Research Promotion Center, Tsukuba Clinical Research and Development Organization, University of Tsukuba, Tsukuba, JPN; 3 Department of Anesthesiology, University of Tsukuba Hospital, Tsukuba, JPN; 4 Department of Obstetrics and Gynecology, Institute of Medicine, University of Tsukuba, Tsukuba, JPN; 5 Department of Health Services Research, Institute of Medicine, University of Tsukuba, Tsukuba, JPN; 6 Laboratory of Mathematical Informatics in Medicine, Institute of Medicine, University of Tsukuba, Tsukuba, JPN; 7 Cybermedicine Research Center, University of Tsukuba, Tsukuba, JPN; 8 Health Services Research and Development Center, University of Tsukuba, Tsukuba, JPN; 9 Department of Biostatistics, Institute of Medicine, University of Tsukuba, Tsukuba, JPN; 10 Center for Artificial Intelligence Research, University of Tsukuba, Tsukuba, JPN

**Keywords:** blood loss, caesarean section, general anaesthesia, total intravenous anaesthesia (tiva), uterine relaxing effect, volatile anaesthesia

## Abstract

Background: Volatile anaesthetics have been suspected to increase intraoperative blood loss during caesarean section by causing uterine relaxation, but evidence from human studies remains inconclusive.

Methods: We conducted a retrospective observational study using a comprehensive perioperative dataset from the University of Tsukuba Hospital. We included 276 caesarean deliveries performed under general anaesthesia between April 2012 and March 2022, comparing patients who received volatile anaesthesia (n = 230) versus total intravenous anaesthesia (TIVA, n = 46). The primary outcome was intraoperative blood loss, and the secondary outcome was the administration of blood transfusion in the operating room. Multivariable regression models were used to adjust for differences in baseline characteristics.

Results: A total of 276 caesarean deliveries were included, with 230 patients in the volatile anaesthesia group and 46 in the TIVA group. The mean blood loss was 1,134.8 mL (SD 992.9 mL) in the volatile group and 1,097.8 mL (SD 764.1 mL) in the TIVA group. After adjustment for covariates, including emergency surgery and neoplasm, no significant difference in blood loss was observed (mean difference [volatile anaesthesia minus TIVA] 182 mL; 95% CI -123.5 to 487 mL; P = 0.244). Blood transfusion was performed in 8.3% of patients in the volatile group and 17.4% in the TIVA group, with no significant difference in the adjusted model (odds ratio 0.54; 95% CI 0.22 to 1.49; P = 0.211).

Conclusion: This study found no significant association between the use of volatile anaesthesia and increased intraoperative blood loss or transfusion during caesarean section.

## Introduction

Maternal mortality during the perinatal period remains a major global concern [1,2], and cesarean sections performed under general anesthesia carry a higher risk than those performed under regional anesthesia [3]. General anesthesia is typically reserved for emergency situations, failures of regional anesthesia, or when contraindications to regional techniques exist [4]. Among general anesthesia options, anesthesiologists may choose either volatile anesthesia or total intravenous anesthesia (TIVA) [4].

Previous experimental studies, including those using animal models and human uterine tissue, have suggested that volatile anesthetics may cause uterine relaxation [5-7]. In contrast, propofol, a commonly used agent in TIVA, has not demonstrated a reduction in uterine tone at clinically relevant concentrations [8]. Based on these findings, some anesthesiologists suspect that volatile anesthesia may lead to increased blood loss during cesarean section compared with TIVA [9-11]. Given that hemorrhage is one of the leading causes of maternal morbidity and mortality during caesarean delivery [12], understanding the potential impact of anesthetic type on bleeding risk is clinically important.

Despite the theoretical concern, there remains a lack of high-quality human studies addressing this issue. A small randomized controlled trial conducted nearly three decades ago (n = 40) found no significant difference in blood loss between the two anesthetic approaches [13]. More recently, a large-scale analysis using instrumental variable methods suggested an association between volatile anesthesia and increased blood loss during delivery [14]. However, in that same study, multivariable regression and propensity score analyses yielded conflicting results. Given the difficulty of directly validating the validity of instrumental variables [15], further investigation using independent data sources that include potential unmeasured confounders is warranted to assess the robustness of the prior findings.

To address this gap, we leveraged a comprehensive perioperative dataset from the University of Tsukuba Hospital that includes detailed intraoperative records [16]. By integrating this with electronic health records and departmental data through patient identifiers, we were able to construct a longitudinal dataset encompassing the full peri-hospitalization period. This allowed us to re-examine the relationship between anesthetic options and blood loss during caesarean section, using a cohort not included in the previous study [14] and incorporating variables that may confound the association. Clarifying this relationship is essential, as confirmation of increased bleeding risk would argue for caution in the use of volatile agents during cesarean section, while a lack of association would support their continued use, especially given their known stability in general anesthesia.

## Materials and methods

This study was approved by the Institutional Review Board (IRB) of the University of Tsukuba Hospital (Approval No. R03-068 and R05-254). Given the retrospective design of this study, the requirement for informed consent was waived. Instead, an opt-out option was made available on our hospital’s website. Following IRB approval and opt-out notification, the study protocol was prospectively registered in the University Hospital Medical Information Network (UMIN) clinical trials registry (UMIN000054654, registered on June 14, 2024).

Study design and data sources

This single-center retrospective observational study was conducted using the perioperative dataset from the University of Tsukuba Hospital. This research dataset was created by linking and de-identifying multiple institutional data sources, including: surgical department data: Fortec ORSYS (Philips Japan, Ltd., Tokyo, Japan); intensive care unit records: Fortec ACSYS and PIMS (Philips Japan, Ltd., Tokyo, Japan); electronic health records (Fujitsu Japan Ltd., Kanagawa, Japan); Diagnosis Procedure Combination (DPC) data, which includes information on admission, discharge, diagnoses, and procedures [17]; physiological test data: PrimeVita® (Nihon Kohden Corporation, Tokyo, Japan); dialysis records: Future Net Web+® (Nikkiso Co., Ltd., Tokyo, Japan).

The dataset includes approximately 70,000 surgeries performed under general, epidural, or spinal anesthesia at the hospital between April 1, 2012, and March 31, 2022. Owing to the linkage across multiple departmental systems, the dataset enables integrated perioperative analysis from admission through discharge. For example, patient demographics and intraoperative records were obtained from the surgical data, outcomes and comorbidities from the DPC data, and laboratory values from the electronic health records. Details of the dataset structure are provided in Appendix 1.

Study population

From the dataset described above, we extracted cases of cesarean delivery performed between April 1, 2012, and March 31, 2022, defined by the procedural codes K898 (caesarean section) or K904 (hysterectomy during pregnancy, i.e., Porro operation). We excluded patients who underwent hysterectomy during the same hospitalization (codes K876, K877, or K904), as well as those who received anesthesia other than general anesthesia. If the same patient underwent multiple cesarean sections under general anesthesia during the study period, each surgery was treated as a separate case.

Exposure definition

Anesthetic technique was determined using entries in the Japanese Society of Anesthesiologists Perioperative Information Management System (JSA PIMS) section of the Vi-pros surgical record. Patients were classified into either the volatile anesthesia group or the TIVA group based on this documentation.

Outcomes

The primary outcome was intraoperative blood loss (ml). The secondary outcome was the administration of blood transfusion (red blood cells, fresh frozen plasma, or platelets) in the operating room.

Covariates

Baseline characteristics were classified into four categories-basic and obstetric factors, comorbidities, surgical features, and laboratory data-based on prior studies [14,18-21]. Disease definitions followed ICD-10 coding, with full definitions provided in Appendix 2. Diagnoses were obtained from the list of comorbidities recorded at admission in the DPC data.

Basic and obstetric factors included age, body mass index (BMI), American Society of Anesthesiologists physical status (ASA-PS) [22], year of delivery (categorized as 2012-2015, 2016-2019, or 2020-2022 to account for potential improvements in surgical technique), placenta previa, placental disorders, multiple pregnancy, previous cesarean delivery or history of uterine surgery, uterine leiomyoma, gestational hypertension, and preeclampsia or eclampsia. Although the presence of placenta previa could be identified from diagnosis codes (O44), severity could not be determined directly. Therefore, a board-certified obstetrician (Mana Obata-Yasuoka) retrospectively reviewed ultrasound images for cases coded as O44, blinded to group allocation. These cases were further classified into (i) anterior vs. posterior placenta (anterior location associated with higher risk) and (ii) placenta previa vs. low-lying placenta (the former being more severe).

Comorbidities included anemia, thrombocytopenia/coagulopathy, hypercoagulable state, asthma, ischemic heart disease, cardiomyopathy, congenital heart disease, valvular heart disease, cerebrovascular disease, diabetes mellitus, liver disease, renal disease, and neoplasm.

Surgical features included urgency of surgery and operative time. Emergency cesarean sections were identified primarily by the procedure code K898-1. For procedures coded as K898-3 (used before 2015 for certain complicated or preterm cases), surgeries were also classified as emergency if the ASA-PS included the emergency modifier “E” or if “emergency cesarean section” was selected in the operative scheduling record in the electronic health system.

Laboratory data included hemoglobin concentration (Hb [g/dl]), platelet count (Plt [103/μl]), prothrombin time (PT [%]), and activated partial thromboplastin time (APTT [sec]). The value closest to the date of surgery within the 90-day preoperative period was used. If no test results were available within this window, the earliest available result on the day of cesarean section was used instead.

Statistical analysis

All statistical analyses were conducted using R (version 4.5.1, R Foundation for Statistical Computing, Vienna, Austria), which is freely available open-source software. All statistical packages and methods used in the present analysis were free to use. Continuous variables were summarized as mean ± standard deviation (SD), and categorical variables as counts and percentages. Patient characteristics’ comparisons were performed using unpaired t-tests [23] for continuous variables and chi-square tests [24] for categorical variables.

For the primary outcome, unadjusted linear regression [25] was used with blood loss as the dependent variable and anesthetic technique as the independent variable. Adjusted models included covariates with a P < 0.05 from the baseline comparison. For the secondary outcome (blood transfusion in the operating room), logistic regression [26] was used similarly, with and without adjustment for the same covariates.

As a post hoc analysis, blood loss was log-transformed, and regression analyses were repeated to assess robustness of the findings.

## Results

**Figure 1:**
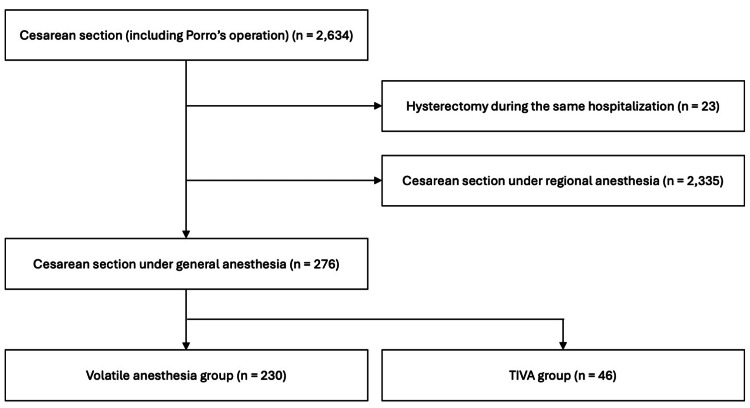
Flow chart of patient selection Abbreviation: TIVA, total intravenous anaesthesia.

From the dataset, 2,634 cases of caesarean section were initially identified. Of these, 23 cases were excluded due to concurrent hysterectomy. An additional 2,335 cases performed under regional anaesthesia were also excluded. As a result, 276 cases were included in the final analysis, comprising 230 patients in the volatile anaesthesia group and 46 patients in the TIVA group (Figure 1).

Patient characteristics are summarized in Table 1. Among basic and obstetric factors, the mean maternal age was similar between groups (33.4 years in the volatile anaesthesia group vs. 32.8 years in the TIVA group; P = 0.529). The gestational age was also comparable (mean 35.8 weeks, SD 4.5 weeks, median 37 weeks, interquartile range [IQR] 34-38 weeks in the volatile anaesthesia group vs. mean 35.7 weeks, SD 3.6 weeks, median 36 weeks, IQR 34-38 weeks in the TIVA group; P = 0.916). Notably, data on gestational age were missing in 48 cases in the volatile anaesthesia group and nine cases in the TIVA group; thus, these calculations were based on complete cases. There were no significant differences in the severity of placenta previa between groups (P = 0.895): no cases of anterior placenta previa were observed in either group; posterior placenta previa was observed in seven patients (3.0%) in the volatile group and two patients (4.3%) in the TIVA group; anterior low-lying placenta in one patient (0.4%) in the volatile group and none in the TIVA group; and posterior low-lying placenta in one patient (0.4%) in the volatile group and none in the TIVA group. Among comorbidities, the prevalence of neoplasms was significantly higher in the TIVA group (8.7%) compared to the volatile anesthesia group (2.6%, P = 0.046). No other comorbid conditions showed statistically significant differences between groups. Regarding surgical characteristics, emergency surgery was more frequent in the volatile anaesthesia group than in the TIVA group (88.3% vs. 65.2%, P < 0.001). Regarding laboratory data related to coagulation, no significant differences were observed between the two groups. In the volatile anesthesia group, 189 patients received sevoflurane only, 44 received desflurane only, and five received both agents; in nine patients, usage records contained errors and the precise amounts could not be determined. The mean total administered volume of sevoflurane was 17.0 mL (SD 12.4 mL, median 15.1 mL, IQR 10.3-22.1 mL), while that of desflurane was 43.7 mL (SD 34.6 mL, median 37.1 mL, IQR 27.8-50.4 mL). In the TIVA group, accurate drug records were available for 45 of 46 patients; all received propofol, with a mean total dose of 717.4 mg (SD 560.0 mg, median 612.5 mg, IQR 428.3-700.0 mg).

**Table 1:** Patient characteristics Abbreviations: APTT, activated partial thromboplastin time; ASA-PS, the American Society of Anaesthesiologists physical status [22]; BMI, body mass index; COPD, chronic obstructive pulmonary disease; Hb, hemoglobin; IQR, interquartile range; Plt, platelet; PT, prothrombin time; TIVA, total intravenous anaesthesia; VA, volatile anaesthesia. Data are presented as mean ± standard deviation or number (%). *P* values and test statistics were derived from t-tests [23] or chi-square tests [24] as appropriate. ^a^Twenty-seven cases were missing in the VA group and two in the TIVA group. ^b^Forty-eight cases were missing in the VA group and nine in the TIVA group. Median gestational ages were 37 weeks (IQR 34–39 weeks) in the VA group and 36 weeks (IQR 34–38 weeks) in the TIVA group. ^c^One case was missing in the VA group and none in the TIVA group. ^d^Two cases were missing in the VA group and none in the TIVA group. ^e^Three cases were missing in the VA group and none in the TIVA group.

	VA group (n = 230)	TIVA group (n = 46)	P value	Test statistic
Basic and obstetric factors				
Age, years	33.4 ± 5.3	32.8 ± 5.2	0.529	–0.631
BMI^a^, kg m^-2^	26.8 ± 5.3	26.7 ± 4.3	0.915	–0.106
ASA-PS				
1	12 (5.2)	1 (2.2)	0.145	0.758
2	46 (20.0)	16 (34.8)		
3	44 (19.1)	11 (23.9)		
4	5 (2.2)	1 (2.2)		
NA	123 (53.5)	17 (37.0)		
Gestational age^b^, week	35.8 ± 4.5	35.7 ± 3.6	0.916	–0.105
Period of delivery				
2012 (April) – 2016 (March)	89 (38.7)	23 (50.0)	0.130	2.048
2016 (April) – 2020 (March)	80 (34.8)	9 (19.6)		
2020 (April) – 2023 (March)	61 (26.5)	14 (30.4)		
Placenta previa (ICD10)	9 (3.9)	2 (4.3)	0.892	0.019
Placenta previa (echo)				
Anterior	0 (0.0)	0 (0.0)	NA	NA
Posterior	7 (3.0)	2 (4.3)	0.653	0.203
Low-lying placenta (echo)				
Anterior	1 (0.4)	0 (0.0)	0.652	0.204
Posterior	1 (0.4)	0 (0.0)	0.652	0.204
Placental disorders	2 (0.9)	1 (2.2)	0.440	0.597
Multiple gestation	22 (9.6)	6 (13.0)	0.480	0.500
Previous caesarean delivery or history of uterine surgery	34 (14.8)	8 (17.4)	0.656	0.198
Uterine leiomyoma	8 (3.5)	1 (2.2)	0.653	0.203
Gestational hypertension	4 (1.7)	0 (0.0)	0.350	0.877
Preeclampsia/eclampsia	45 (19.6)	4 (8.7)	0.082	3.039
Comorbidities				
Anemia	29 (12.6)	6 (13.0)	0.936	0.199
Thrombocytopenia/coagulopathy	6 (2.6)	2 (4.3)	0.525	0.405
Hypercoagulable state	2 (0.9)	0 (0.0)	0.518	0.418
Asthma	3 (1.3)	1 (2.2)	0.656	0.199
Ischemic heart disease	2 (0.9)	0 (0.0)	0.518	0.418
Valvular heart disease	5 (2.2)	2 (4.3)	0.397	0.720
Cerebrovascular disease	2 (0.9)	0 (0.0)	0.518	0.418
Diabetes mellitus	45 (19.6)	6 (13.0)	0.304	1.061
Liver disease	4 (1.7)	0 (0.0)	0.350	0.877
Renal disease	1 (0.4)	1 (2.2)	0.208	1.590
Neoplasm	6 (2.6)	4 (8.7)	0.046	4.006
Surgical features				
Emergency surgery	203 (88.3)	30 (65.2)	< 0.001	15.221
Operative time (min)	69.2 ± 56.3	81.8 ± 64.9	0.222	1.225
Laboratory data				
Hb^c^, g/dl	11.0 ± 1.3	10.8 ± 1.4	0.361	–0.914
Plt^c^, 10^3^/μl	216.1 ± 70.3	209.1 ± 83.0	0.592	-0.537
PT^d^ (%)	119.0 ± 23.3	114.7 ± 17.1	0.141	–1.475
APTT^e^ (sec)	31.3 ± 8.3	33.2 ± 15.8	0.424	0.801

**Figure 2:**
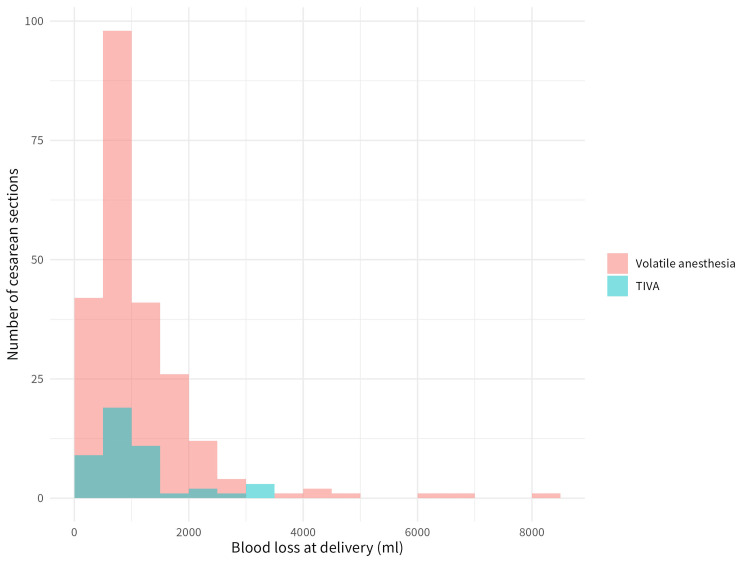
Histograms of blood loss at delivery for the total intravenous and volatile anaesthesia groups Abbreviation: TIVA, total intravenous anaesthesia.

Histograms of intraoperative blood loss are shown in Figure 2. The mean blood loss was 1,134.8 mL in the volatile anaesthesia group (SD 992.9 mL; median 850.0 mL; IQR 576.3-1,392.5 ml) and 1,097.8 mL in the TIVA group (SD 764.1 ml; median 878.5 ml; IQR 642.5-1,329.0 ml). Intraoperative blood transfusion was performed in 19 of 230 patients (8.3%) in the volatile anaesthesia group and in 8 of 46 patients (17.4%) in the TIVA group.

Results of the regression analyses are summarized in Table 2. In the adjusted model, neoplasm and emergency surgery status were included as covariates. In the primary analysis with intraoperative blood loss as the outcome, the unadjusted model showed no significant difference between groups (mean difference 37 ml [positive values indicate greater blood loss in the volatile anaesthesia group]; 95% confidence interval [CI] -267 to 341 ml; P = 0.811). Similarly, the adjusted model also showed no significant difference (mean difference 182 ml; 95% CI -123.5 to 487 ml; P = 0.244). For the secondary outcome of intraoperative transfusion, the unadjusted model showed no statistically significant difference between groups (odds ratio [OR] 0.43 [values <1 indicate fewer transfusions in the volatile anaesthesia group]; 95% CI 0.18 to 1.10; P = 0.063). The adjusted model likewise revealed no significant difference (OR 0.54; 95% CI 0.22 to 1.49; P = 0.211).

**Table 2:** Differences in blood loss at delivery and odds ratios of blood transfusions in operating room Abbreviations: CI, confidence interval; OR, odds ratio. For continuous outcomes, comparisons were made using t-tests derived from linear regression models [25], whereas binary outcomes were evaluated using Wald tests based on logistic regression models [26]. ^a^Positive values for blood loss difference indicate greater blood loss in the volatile anaesthesia (VA) group (i.e., TIVA minus volatile anaesthesia). ^b^OR represent the odds in the volatile anaesthesia group divided by those in the TIVA group. ^c^Model adjusted for background factors with P value below 0.05 (“neoplasm” and “emergency surgery”)

	Blood loss at delivery				Blood transfusions in operating room		
	Difference^a^ (ml, 95% CI)	P value	Test statistic		OR^b^ (95% CI)	P value	Test statistic
Crude	37.0 (-266.6 to 340.6)	0.811	0.239		0.43 (0.18 to 1.10)	0.063	–1.859
Adjusted^c^	182.0 (-123.5 to 487.4)	0.243	1.168		0.54 (0.22 to 1.49)	0.211	–1.250

As a post hoc analysis, intraoperative blood loss was log-transformed, and results are presented in Appendices 3 and 4. Similar to the non-transformed analysis, no significant differences were observed between groups in either model. In the unadjusted model, the blood loss ratio (volatile anaesthesia group relative to TIVA group) was 0.98 (95% CI 0.80 to 1.22; P = 0.885), and in the adjusted model, the ratio was 1.08 (95% CI 0.87 to 1.34; P = 0.478).

## Discussion

Using a comprehensive perioperative dataset from the University of Tsukuba Hospital, this study found no significant association between volatile anaesthesia and increased blood loss at delivery. A post hoc analysis using log-transformed blood loss values yielded consistent results, with no significant difference between the groups. For the secondary outcome of intraoperative blood transfusion, no significant difference was observed between the groups.

To the best of our knowledge, no previous clinical studies have compared the type of general anaesthesia and blood loss during caesarean section using a relatively large and detailed dataset. Although the number of cases in the present study is smaller than the previous large-scale database study [14], our analysis benefits from the availability of detailed clinical data. This allowed for more precise comparisons between groups, including the severity of placenta previa and preoperative laboratory values - an aspect not addressed in the previous study. Observational studies are particularly valuable in this context, as it is generally difficult to conduct randomized controlled trials for urgent caesarean sections performed under general anaesthesia. Moreover, given the large variability in blood loss, the use of retrospective data is appropriate and necessary for meaningful analysis.

Importantly, our results suggest that the choice of general anaesthetic agent during caesarean section may not have a substantial impact on blood loss. This finding may help reassure anaesthesiologists that the use of volatile anaesthetics during caesarean section does not necessarily increase bleeding risk and may reduce unnecessary concern regarding their use in clinical practice.

Our findings were inconsistent with the hypotheses proposed in previous preclinical studies [5,6,8] and clinical investigations [9-11,14] suggesting that volatile anaesthesia may increase blood loss. However, a basic study using human myometrial tissue obtained during caesarean section demonstrated that propofol can inhibit uterine contractions in a concentration-dependent manner [27]. These findings may suggest that, at clinically relevant concentrations, volatile and intravenous anaesthetics exert a comparable degree of uterine relaxation.

While some differences in patient characteristics were observed between the two groups, others were more similar than expected. For instance, the TIVA group had a higher proportion of patients with neoplasms, suggesting that these cases may have involved non-standard surgical planning or anaesthetic considerations. In contrast, the higher frequency of emergency caesarean sections in the volatile anaesthesia group likely reflects its preference in urgent situations, where rapid and stable induction and maintenance of anaesthesia are required. On the other hand, factors such as the severity of placenta previa and preoperative laboratory data - both closely related to bleeding risk - were comparable between groups. This suggests that few anaesthesiologists select volatile anaesthesia or TIVA primarily based on anticipated bleeding risk.

Several limitations should be acknowledged. First, the number of caesarean sections performed under general anaesthesia at our institution was relatively small, raising the possibility of type II error. Second, the primary outcome - blood loss at delivery - is susceptible to measurement error. However, this study was conducted at a single institution, which may reduce inter-facility variability in measurement methods. Third, in this study, the proportion of cases in which TIVA was selected as the method of general anaesthesia for caesarean section was extremely low. A previous study reported inter-facility variation in the choice of general anaesthetic agents for caesarean sections under general anaesthesia in Japan [14]. In the present study, information was not available regarding the reasons for the use of either volatile anaesthesia or TIVA, and therefore, the rationale behind this distribution remains unclear. Accordingly, we cannot rule out the possibility that unmeasured confounding factors, including potential differences between groups, may have influenced the results. Fourth, important variables such as ASA-PS had a high proportion of missing values, possibly due to omissions in documentation in urgent situations. This may have made the comparison of patient backgrounds inaccurate and introduced bias in the comparison of outcomes. Fifth, there is a possibility that some patients may have been converted from volatile anesthesia to TIVA due to excessive intraoperative bleeding. In such cases, because volatile anesthetics were used at any point during the procedure, these patients would have been categorized into the volatile anesthesia group. This could have introduced a bias toward higher estimated blood loss in the volatile anesthesia group. Therefore, a prospective study would be needed to more accurately estimate differences in blood loss between the two anesthetic techniques. Lastly, because our institution is a tertiary academic hospital, the case mix may be more complex and severe than in general hospitals, limiting the generalizability of our findings. To address these limitations, future multicenter studies using similarly detailed data are warranted.

## Conclusions

In conclusion, in this detailed comparison using the University of Tsukuba Hospital perioperative dataset, we found no evidence of an association between volatile anesthesia and increased blood loss during cesarean section. These findings suggest that, based on the current evidence, clinicians may not need to place substantial emphasis on anesthetic choice solely from the perspective of hemorrhage risk in this setting. However, further large-scale studies incorporating more detailed and granular perioperative data are warranted to validate these results and to determine whether specific patient subgroups might benefit from tailored anesthetic strategies.

## Figures and Tables

**Figure 3:**
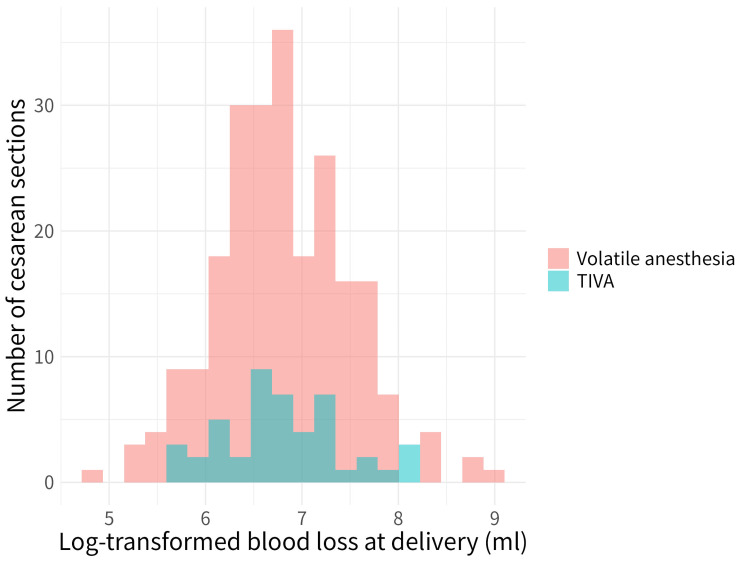
Log-transformed histograms of blood loss at delivery for the volatile and total intravenous anaesthesia groups Abbreviation: TIVA, total intravenous anaesthesia.

## Appendices

Appendix 1 

**Table 3:** Data source details Abbreviations: ASA-PS, American Society of Anaesthesiologists physical status [22]; BMI, body mass index; DPC, Diagnosis Procedure Combination.

Variables		Data sources
Study population		
	Caesarean section	DPC data
	General anaesthesia	ORSYS (anaesthesia records)
Definition of the Groups		
	Anaesthetic technique	ORSYS (anaesthesia records)
Outcomes		
	Blood loss	ORSYS (anaesthesia records)
	Blood transfusion	ORSYS (anaesthesia records)
Patient characteristics		
	Age, BMI, ASA-PS, period of delivery	ORSYS (preoperative consultation in anaesthesiology)
	Obstetric factors and comorbidities	DPC data
	Emergency surgery	DPC data or ORSYS (preoperative consultation in anaesthesiology)
	Operative time	ORSYS (anaesthesia records)
	Laboratory data	Electronic health records (Fujitsu Japan Ltd.)

Appendix 2  

**Table 4:** ICD-10 definitions Abbreviation: ICD-10, International Classification of Diseases, 10th Edition.

Characteristics of women on admission	ICD-10 codes
Placenta previa	O44.x
Placental disorders	O43.x
Multiple gestation	O30.x
Previous caesarean delivery or history of uterine surgery	O34.2
Uterine leiomyoma	D25.x, O34.1
Infection of the amniotic sac and membranes	O41.1
Pregnancy-induced hypertension	
Gestational hypertension	O13, O16
Preeclampsia/eclampsia	O11, O14.x, O15.0
Anemia	D46.x, D50.x, D51.x, D52.x, D53.x, D55.x, D56.x, D58.x, D59.x, D60.x, D62, D63.x, D64.x, O99.0
Thrombocytopenia/coagulopathy	D61.x, D66, D67, D68.0–D68.4, D68.9, D69.0, D69.1, D69.3–D69.9, M31.1
Hypercoagulable state	D68.8
Asthma	J45.x, J46
Ischemic heart disease	I20–25.x
Cardiomyopathy	I42.0
Congenital heart disease	Q20.x, Q21.x, Q22.x, Q23.x, Q24.x, Q25.x, Q26.0, Q26.2, Q26.3, Q26.4
Valvular heart disease	I05–08.x, I34–37.x, I39.0–39.4
Cerebrovascular disease	I60–69.x
Diabetes mellitus	E10.x, E11.x, E12.x, E13.x, E14.x, O24.x
Liver disease	B18.x, K70–77.x
Renal disease	I12.0, I13.x, I15.1, N00–19, N26–29, N99.0
Neoplasm	C00–C96.x

Appendix 3  

Appendix 4  

**Table 5:** Comparison of log-transformed blood loss at delivery between the volatile and total intravenous anaesthesia groups Abbreviations: CI, confidence interval; OR, odds ratio. P values and test statistics were derived from linear regression models (t-tests) [25]. ^a^The blood loss at delivery in the volatile anaesthesia group was divided by the blood loss at delivery in the TIVA group. ^b^Model adjusted for background factors with P value below 0.05 (“neoplasms” and “emergency surgery”)

	Blood loss at delivery		
	TIVA-to-volatile anaesthesia ratio^a^ (95% CI)	P value	Test statistics
Crude	0.98 (0.80 to 1.22)	0.885	–0.145
Adjusted^b^	1.08 (0.87 to 1.34)	0.478	0.710
